# Altered Ratio of D1 and D2 Dopamine Receptors in Mouse Striatum Is Associated with Behavioral Sensitization to Cocaine

**DOI:** 10.1371/journal.pone.0011038

**Published:** 2010-06-09

**Authors:** Dawn Thompson, Lene Martini, Jennifer L. Whistler

**Affiliations:** 1 Ernest Gallo Clinic and Research Center, University of California San Francisco, Emeryville, California, United States of America; 2 Department of Neurology, University of California San Francisco, San Francisco, California, United States of America; University of Queensland, Australia

## Abstract

**Background:**

Drugs of abuse elevate brain dopamine levels, and, *in vivo*, chronic drug use is accompanied by a selective decrease in dopamine D2 receptor (D2R) availability in the brain. Such a decrease consequently alters the ratio of D1R∶D2R signaling towards the D1R. Despite a plethora of behavioral studies dedicated to the understanding of the role of dopamine in addiction, a molecular mechanism responsible for the downregulation of the D2R, *in vivo*, in response to chronic drug use has yet to be identified.

**Methods and Findings:**

Ethics statement: All animal work was approved by the Gallo Center IACUC committee and was performed in our AAALAC approved facility. In this study, we used wild type (WT) and G protein coupled receptor associated sorting protein-1 (GASP-1) knock out (KO) mice to assess molecular changes that accompany cocaine sensitization. Here, we show that downregulation of D2Rs or upregulation of D1Rs is associated with a sensitized locomotor response to an acute injection of cocaine. Furthermore, we demonstrate that disruption of GASP-1, that targets D2Rs for degradation after endocytosis, prevents cocaine-induced downregulation of D2Rs. As a consequence, mice with a GASP-1 disruption show a reduction in the sensitized locomotor response to cocaine.

**Conclusions:**

Together, our data suggests that changes in the ratio of the D1R∶D2R could contribute to cocaine-induced behavioral plasticity and demonstrates a role of GASP-1 in regulating both the levels of the D2R and cocaine sensitization.

## Introduction

Drugs of abuse cause long lasting alterations in dopaminergic neurotransmission. Dopamine mediates its effects through its action at five distinct receptors (D1, D2, D3, D4 and D5) belonging to the G protein-coupled receptor (GPCR) superfamily. These receptors can be subdivided into two groups: the D1-like (D1 and D5), which are coupled to the stimulatory G proteins G_s_ and G_olf_, and the D2-like (D2, D3, D4), which are coupled to the inhibitory G proteins G_i/o_. Disruptions in dopaminergic signaling have been implicated in many neurological disorders, including Parkinson's disease, schizophrenia, Tourette's syndrome, depression and addiction. Importantly, studies using positron emission topography (PET) have consistently shown that nicotine [1], heroin [2], alcohol [3], [4], methamphetamine [5] and cocaine [6], [7] abuse are accompanied by a decrease in striatal D2 receptor (D2R) availability. Indeed, drug-induced loss of D2R is apparent across multiple species including rodents [8], primates [6] and humans [2]-[5], [7]. Furthermore, studies in drug naïve non-human primates suggest that D2R availability is predictive of future drug seeking behavior [6]. In addition, in socially-housed macaques, D2R availability increases in subjects that achieve social dominance, and is lower in the subjects that become subordinate [9]. In this setting, the subordinate subjects with low D2R availability exhibit significantly greater cocaine seeking than the dominant subjects with high D2R availability. Together, these data suggest that low D2R levels contribute to drug-seeking behavior, whether those levels are inherently low or are decreased due to environmental influences. However, the molecular mechanisms that mediate loss of D2R *in vivo* remain unclear.

Dopamine receptor mediated signaling is extensively regulated by numerous processes, including, endocytosis, whereby agonist activated receptors are rapidly silenced by removal from the cell surface to an endocytic compartment. Both the D1 and the D2 receptors can be internalized after activation by dopamine [10]. However, one important distinction between D2R and D1R is receptor fate following endocytosis [10]. Specifically, the D1R is rapidly recycled and, thus, returns to the cell surface where it may bind ligand once again. In contrast, the D2R is targeted for degradation in the lysosome through its interaction with the GPCR-associated sorting protein (GASP-1) [11]. Thus, we hypothesized that downregulation of D2Rs in response to cocaine, or other drug exposure that increase dopamine levels, could arise from a GASP-mediated postendocytic degradation of D2Rs. By extension, we hypothesized, that disruption of GASP-1 would interfere with cocaine-induced behavioral plasticity by preventing D2R downregulation.

Repeated exposure to psychostimulants promotes a progressive and long lasting enhancement of drug-induced locomotor stimulation [12]–[14]. This phenomenon of “behavioral sensitization” is thought to underlie aspects of addiction [14]. Many electrophysiological and cellular neuroadaptations occur during repeated drug use [15], [16]. The precise role of dopamine in these changes is still being vigorously debated. Nevertheless, all drugs of abuse increase dopamine levels and, thereby, activity at the dopamine receptors. Accordingly, here we examined the hypothesis that cocaine-induced behavioral sensitization is mediated by an alteration in the balance of excitatory D1R versus inhibitory D2R signal transduction due to GASP-1 mediated degradation of D2R under conditions of high dopamine tone. We demonstrate that both D2R downregulation and sensitization to repeated cocaine exposure is attenuated in GASP-1 KO mice. Furthermore, we found that either upregulation of D1Rs or downregulation of D2Rs is associated with behavioral consequences in response to acute cocaine exposure.

## Results

**Figure 1:**
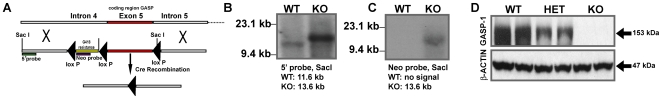
Generation of GASP-1 knockout mice. **A**. Targeting vector design for generating GASP-1 KO mice. A cassette expressing the G418 resistance gene flanked by lox P sites was inserted into the intron upstream of the GASP-1 open reading frame (ORF) (intron 4) and a third loxP site was inserted in the intron downstream of the GASP ORF (intron 5). ES cells from C57/Bl6 mice were transfected with this vector. Properly targeted clones (see B, C) were transfected with Cre-recombinase and blastocysts from clones in which the GASP-1 ORF was disrupted were implanted into C57/BL6 females. B, C. Southern blotting analysis identified homologous recombination and single insertion using **B.** 5′ and **C.** Neo probes. **D.** Immuno-detection of GASP-1 in KO mice. Whole brain lysates from WT, HET and KO mice were prepared, separated by SDS-PAGE, transferred to nitrocellulose and immunoblotted for GASP-1 using a GASP specific antibody (upper panel) or β-Actin (lower panel) to control for protein loading.

To examine whether GASP-1 influenced D2R responses *in vivo*, mice where the GASP-1 gene had been deleted were generated (Fig. 1). Primary ES screening was performed by Southern (DNA) blotting. A knock-in clone was identified as positive for homologous recombination (with a single Neo integration) using a probe to the 5′ region of the GASP-1 gene (Fig. 1B) and a Neo probe (Fig. 1C). These ES cells were then transfected with Cre-recombinase and clones were identified in which exon 5 of the GASP-1 gene, which encodes the entire open reading frame, had been excised. These ES cells were used to generate mice with a disruption of the GASP-1 gene. Mice deficient in GASP-1 expression appeared healthy and indistinguishable from wild type (WT) littermates. To confirm disruption of GASP-1 expression, brain lysates from WT, heterozygous (HET) and KO mice were immunoblotted for GASP-1 (Fig. 1D). GASP-1 immunoreactivity was absent in GASP-1 KOs and was reduced in HETs. Since the gene encoding GASP-1 is on the X chromosome, no HET males were produced.

There was no difference in locomotor response between male WT and KO or female WT, HET or KO mice when placed in a novel environment, nor any differences observed across genotype and sex when mice were tested on an accelerod (data not shown), indicating that KO mice have no gross motor coordination abnormalities or other motor impairments that might interfere with responses to novelty. All subsequent experiments were performed on male WT and KO littermates, 8 to 12 weeks old.

**Figure 2:**
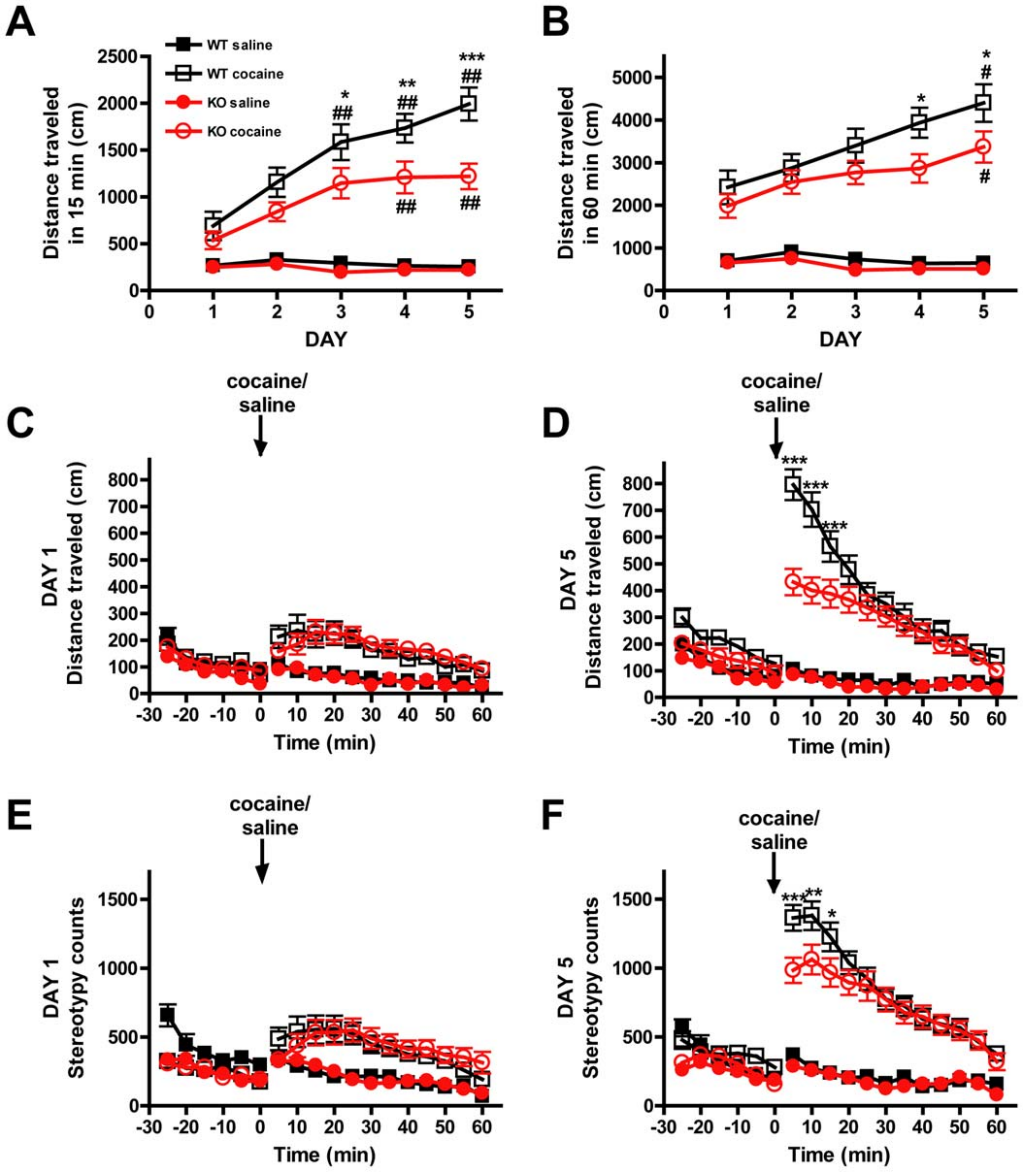
GASP-1 knockout mice exhibit reduced cocaine sensitization. **A-D.** Cocaine sensitization in WT and GASP-1 KO mice. Mice were injected daily (IP) with saline (WT n = 27, GASP-1 KO n = 25) or cocaine (15 mg/kg, WT n = 29, GASP-1 KO n = 30) and the distance traveled reported for **A.** 15 min or **B.** 60 min post-injection. WT mice exhibited a sensitized locomotor response to cocaine by day 3 (WT cocaine, day 3 versus day 1, ##p<0.01 as analyzed by one-way ANOVA followed by Bonferroni multiple comparison tests between corresponding days), while GASP-1 KOs developed a significantly lower sensitized response within this first 15 min by day 4 (GASP-KO cocaine, day 4 versus day 1 ##p<0.01 as analyzed by one-way ANOVA followed by Bonferroni multiple comparison tests between corresponding days). GASP-1 KO mice showed significantly reduced sensitized locomotor response to cocaine compared to WT mice on all days (GASP-1 KO cocaine versus WT cocaine where *p<0.05, **p<0.01 and ***p<0.001 as analyzed by two-way ANOVA followed by Bonferroni multiple comparison tests between corresponding days). GASP-1 KO mice showed reduced locomotor responsiveness to cocaine after repeated treatment both 15 min (**C.** ***p<0.001) and 60 min post-injection (**D.** *p<0.05). **E, F.** Stereotypic behaviors of WT and GASP-1 KO mice after 1 (**E**) and 5 (**F**) days of cocaine exposure. On day 5 there were significant differences in stereotypy at 5, 10 and 15 min (GASP-1 KO cocaine versus WT cocaine where ***p<0.001, **p<0.01 and *p<0.05 respectively as analyzed by two-way ANOVA followed by Bonferroni multiple comparison tests between corresponding days) post-injection between cocaine treated genotypes. Data is presented as mean ± S.E.M.

We next examined whether disruption of GASP-1 altered behavioral responses to cocaine. Acute cocaine sensitivity and development of behavioral sensitization to the locomotor activating effects of cocaine (15 mg/kg, IP for 5 days) was investigated in both WT and GASP-1 KO mice. There was no significant difference in the locomotor activity of WT and GASP-1 KO to acute cocaine treatment after 15 min (Fig. 2A, day 1) or 60 min (Fig. 2B, day 1) post-injection, or at any point over the remainder of the time course (Fig. 2C, day 1). However, GASP-1 KO mice exhibited significantly reduced sensitization that also developed more slowly compared to WT mice. Specifically, GASP-1 KO mice showed significantly reduced locomotor activity compared to WTs during the first 15 min post injection on days 3, 4 and 5 (Fig. 2A, days 3, 4 and 5, *p<0.05, **p<0.01 and ***p<0.001, WT versus GASP-1 KO). GASP-1 KOs also exhibited significantly reduced locomotor activity for the full 60 min time course post-injection compared to WTs on days 4 and 5 (Fig. 2B, days 4 and 5, *p<0.05, WT versus GASP-1 KO). In addition, while WT mice developed a sensitized response by day 3 (Fig. 2A, day 3, ##p<0.01 versus day 1), GASP-1 KO mice did not develop sensitization until day 4 (Fig. 2A, day 4, ##p<0.01 versus day 1). Although by day 4, both WT and GASP-1 KO mice displayed a sensitized response, the GASP-1 KO response was significantly blunted on all days (Fig. 2B,D *p<0.05, **p<0.01 and ***p<0.001, WT versus GASP-1 KO). Finally, while there was no difference in stereotypic behavior between genotypes following their initial exposure to cocaine (Fig. 2E), WT mice developed significantly higher stereotypic behaviors when compared to GASP-1 knockouts (Fig. 2F, ***p<0.001, **p<0.01 and *p<0.05, WT versus GASP-1 KO). These data suggest that the development of locomotor sensitization to repeated cocaine was significantly attenuated in GASP-1 KO mice.

**Figure 3:**
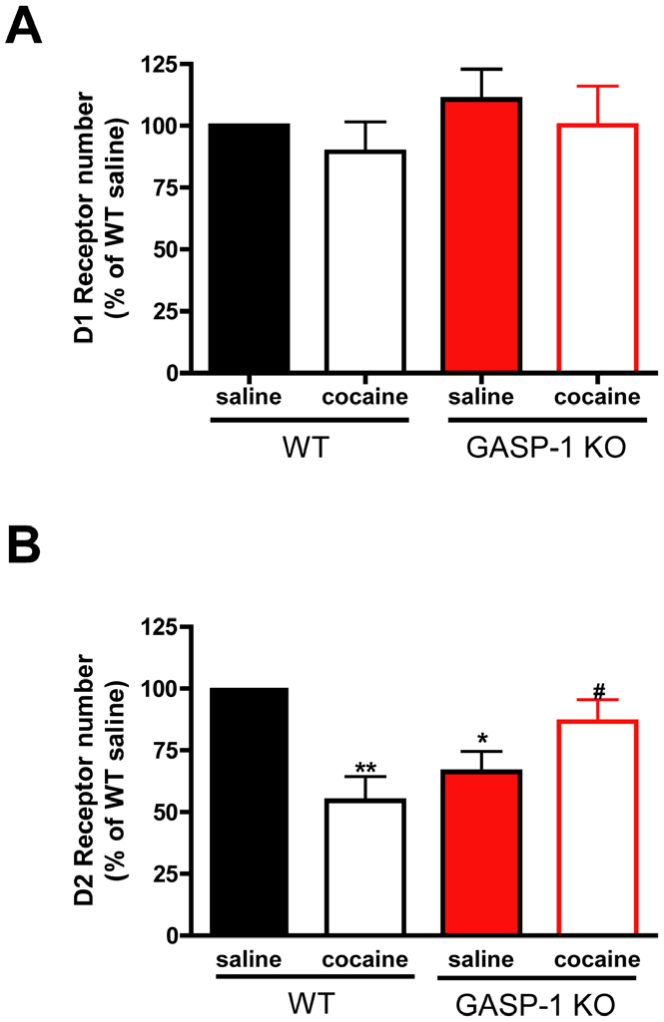
Reduced D2 receptor number in WT mice treated with cocaine. **A.** D1R number in striatal membranes was not affected by cocaine treatment in either genotype when assessed by saturation binding using [^3^H]-SCH23390. **B.** Cocaine treatment reduced D2R number in WT mice (**p<0.01 WT cocaine versus WT saline). Saline treated GASP-1 KO showed reduced D2R number (GASP-1 KO saline versus WT saline where *p<0.05) and D2R number was increased in cocaine treated GASP-1 KO mice (#p<0.05 GASP-1 KO cocaine versus GASP-1 KO saline). D2R number was assessed by saturation binding using [^3^H]-raclopride. Data shown are normalized to saline treated groups and are from three independent radioligand binding experiments, performed in duplicate, with at least eight mice per group.

We next examined whether this paradigm of repeated cocaine administration altered dopamine receptor number. Saturation radioligand binding on striatal membranes using [^3^H]-SCH23390 revealed no significant change in D1R number in either WT or GASP-1 KO mice following cocaine sensitization (Fig. 3A and Table 1), consistent with the ability of this receptor to recycle following endocytosis [10]. Repeated cocaine treatment led to a 50% decrease in D2Rs (Fig. 3B) in WT mice when compared to saline treated mice as observed by a reduction in [^3^H]-Raclopride binding (Fig. 3B and Table 1, **p<0.01, WT cocaine versus WT saline). In contrast, there was an increase in D2R number in GASP-1 KO mice treated with cocaine (#p<0.05, GASP-1 KO cocaine versus GASP-1 KO saline). Interestingly, prior to any treatment GASP-1 KO mice had reduced D2R numbers compared to WT mice (*p<0.05 WT saline versus GASP-1 KO saline). Nevertheless, the net effect of cocaine treatment in WT mice is a decrease in the ratio of DR2∶D1R (Table 1, 0.97 in naïve versus 0.62 in cocaine treated mice), while in GASP-KO mice the ratio of D2R∶D1R is increased (Table 1, 0.58 in naïve GASP-1 KO mice versus 0.74 in cocaine treated mice). Together, these data suggest that GASP-1 may mediate the degradation of the D2R in response to repeated cocaine. They also suggest that a change in the ratio of D2R∶D1R during cocaine treatment, rather than the absolute ratio *per se*, influences the expression of behavioral sensitization.

**Table 1:** Bmax values and D2R:D1R ratios of WT and KO mice saline or cocaine treated.

		Bmax fmol/µg	
		WT	KO
**D1 receptor**			
saline		4.141±0.26	4.432±0.67
cocaine		3.571±0.56	4.100±0.67
**D2 receptor**			
saline		4.013±1.47	2.588±0.72
cocaine		2.218±0.03	3.049±0.80
**D2R:D1R ratio**			
saline		0.97	0.58
cocaine		0.62	0.74

Bmax values were calculated from at least three independent radioligand binding experiments performed in duplicate with pooled striata from at least 8 mice per group. Statistical analyses are shown in Figure 3.

**Figure 4:**
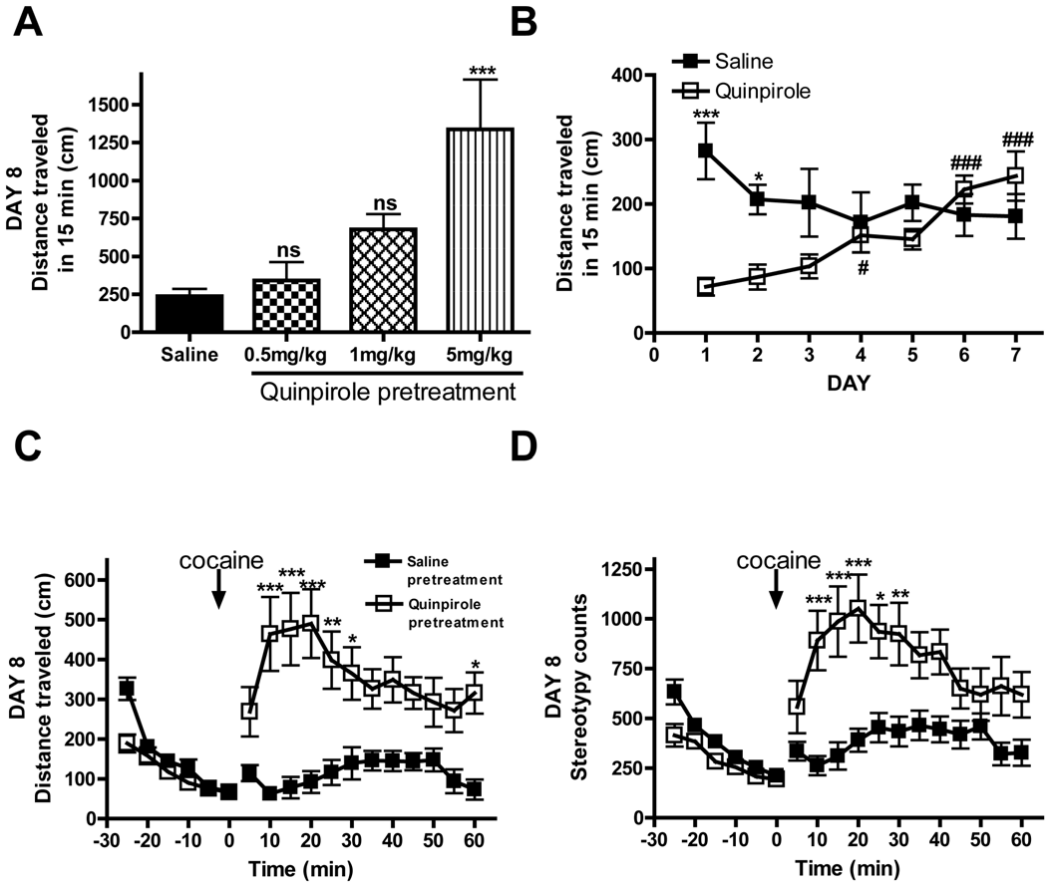
Repeated quinpirole treatment augmented cocaine-induced behavioral responding. **A,** Dose response of quinpirole pretreatment. WT C57/BL6 mice were injected daily (IP) with saline or one of three doses of quinirole (0.5, 1 or 5 mg/kg n = 8 per group) for 7 days. On day 8 all mice were injected (IP) with 20 mg/kg cocaine and their locomotor activity recorded. **B.** Mice become tolerant to the locomotor-suppressing effects of quinpirole. WT C57/BL6 mice were injected daily (IP) with saline (n = 24) or quinpirole (5 mg/kg, n = 24) and their locomotor activity recorded once per day for 7 days. On days 1 and 2, quinpirole reduced locomotor activity when compared to saline treated animals (***p<0.001 and *p<0.05 respectively). WT mice became tolerant to the locomotor inhibitory effects of quinpirole on day 4 and remained so for days 6 and 7 compared to day 1 (#p<0.05 and ###p<0.001 respectively).** C, D.** Repeated quinpirole pre-treatment augments locomotor response to acute cocaine. On day 8, mice from (**B**) were injected with cocaine (IP, 20 mg/kg) and their locomotor activity (**D**) and stereotypy (**E**) recorded. Mice pretreated with repeated quinpirole showed enhanced locomotor and stereotypic responses to acute cocaine compared to saline treated mice (***p<0.001, **p<0.01 and *p<0.05). Data was analyzed by either one or two-way ANOVA with Bonferroni multiple comparison tests and presented as mean ± S.E.M.

To directly examine the role of D2R downregulation in mediating locomotor sensitization to cocaine, WT mice were pre-treated for 7 days with either saline, or the D2R-like agonist quinpirole (IP, 0.5, 1 or 5 mg/kg), which has been shown to promote endocytosis and downregulation of D2Rs both *in vitro* and *in vivo* (10, 17). On day 8, all groups were injected with cocaine (IP, 20 mg/kg) and their locomotor activity was examined. Only mice pre-treated with the highest (5 mg/kg) dose of quinpirole showed significantly higher rates of locomotion in response to acute cocaine when compared to saline treated mice (Fig. 4A, ***p<0.001 versus saline pretreatment) and a significant decrease in D2R number (data not shown). For this reason, the 5 mg/kg dose was used in all subsequent experiments.

WT mice were treated once per day for 7 days with 5 mg/kg quinpirole and locomotion was monitored each day. On days 1 and 2, quinpirole decreased the locomotor responses of WT mice when compared to saline treated mice (Fig. 4B, ***p<0.001 and *p<0.05 respectively), which is consistent with previous reports of the locomotor inhibitory effects of D2R agonists [17], [18]. However, by day 3, there was no longer a significant effect of quinpirole on locomotion compared to saline (Fig. 4B). In addition, by day 4, the locomoter response to quinpirole was significantly reduced compared to the effect on day 1, suggesting that the mice had become tolerant to the locomotor-supressing effects of this drug (Fig. 4B, #p<0.05 qunipirole day 1 versus day 4, ###p<0.001 day 1 versus days 6 and 7).

On day 8, all mice (both saline and quinpirole treated) were injected with cocaine (IP, 20 mg/kg). Consistent with our hypothesis that D2R downregulation contributes to cocaine sensitization, mice pretreated with quinpirole showed significantly greater cocaine-induced locomotor activity (Fig. 4C) and stereotypic behavior (Fig. 4D) compared to mice pretreated with saline (***p<0.001, **p<0.01 and *p<0.05). Saline treated mice in this experiment showed reduced locomotion in response to acute cocaine compared to naïve mice in the previous experiment (Fig. 2). This can likely be attributed to a loss in novelty of the locomotor chamber environment in this but not the previous experiment. Here, mice were exposed to the locomotor boxes for two habituation sessions and then each treatment day to monitor tolerance to quinpirole (Fig. 4B), whereas mice in Figure 2 experienced only two habituation sessions in the locomotor box before the cocaine treatment day. Despite this loss of novelty, quinpirole pre-treatment induced significantly higher locomotor activity to acute cocaine compared to saline pretreatment (Fig. 4C, ***p<0.001, **p<0.01 and *p<0.05).

Finally, radioligand binding in striatal membranes obtained from the mice from Figure 4 after day 8, demonstrated that quinpirole pretreatment promoted downregulation of D2Rs when compared to saline treated mice, while D1R number was unchanged, thus shifting the D2R∶D1R ratio from 0.99 to 0.56 (Table 2, *p<0.05 quinpirole pretreated versus saline pretreated).

**Table 2:** Bmax values and D2R:D1R ratios of WT mice pretreated with quinpirole or aripiprazole.

	Bmax fmol/µg
**D1 receptor**	
Saline	3.411±0.32
Vehicle	3.423±0.34
Quinpirole	3.577±0.14
Aripiprazole	4.295±0.28 *
**D2 receptor**	
Saline	3.361±0.77
Vehicle	3.441±0.11
Quinpirole	2.108±0.72 *
Aripiprazole	2.720±0.47
**D2R:D1R ratio**	
Saline	0.99
Vehicle	1.01
Quinpirole	0.59
Aripiprazole	0.63

Bmax values were calculated from three independent radioligand binding experiments performed in duplicate with 8 mice per group where quinpirole and aripiprazole were compared against their controls (saline and vehicle respectively, *p<0.05).

Recent evidence has suggested that many clinically important antipsychotics behave as inverse agonists at the D2R rather than neutral antagonists as first thought [19]. One clinically important dopamine receptor drug, aripiprizole, is a partial agonist, rather than an inverse agonist/neutral antagonist. Unlike the full agonists quinpirole and dopamine, aripiprizole does not recruit β-arrestin 2 [20] and hence does not internalize the D2R *in vitro* [21]. Furthermore, *in vivo*, aripiprazole was found to attenuate cocaine self-administration and did not support self-administration when given alone [22]. Therefore, we hypothesized the failure of aripiprazole to internalize D2Rs would reduce the likelihood of D2R downregulation and would therefore not produce a sensitized locomotor response to acute cocaine exposure.

**Figure 5:**
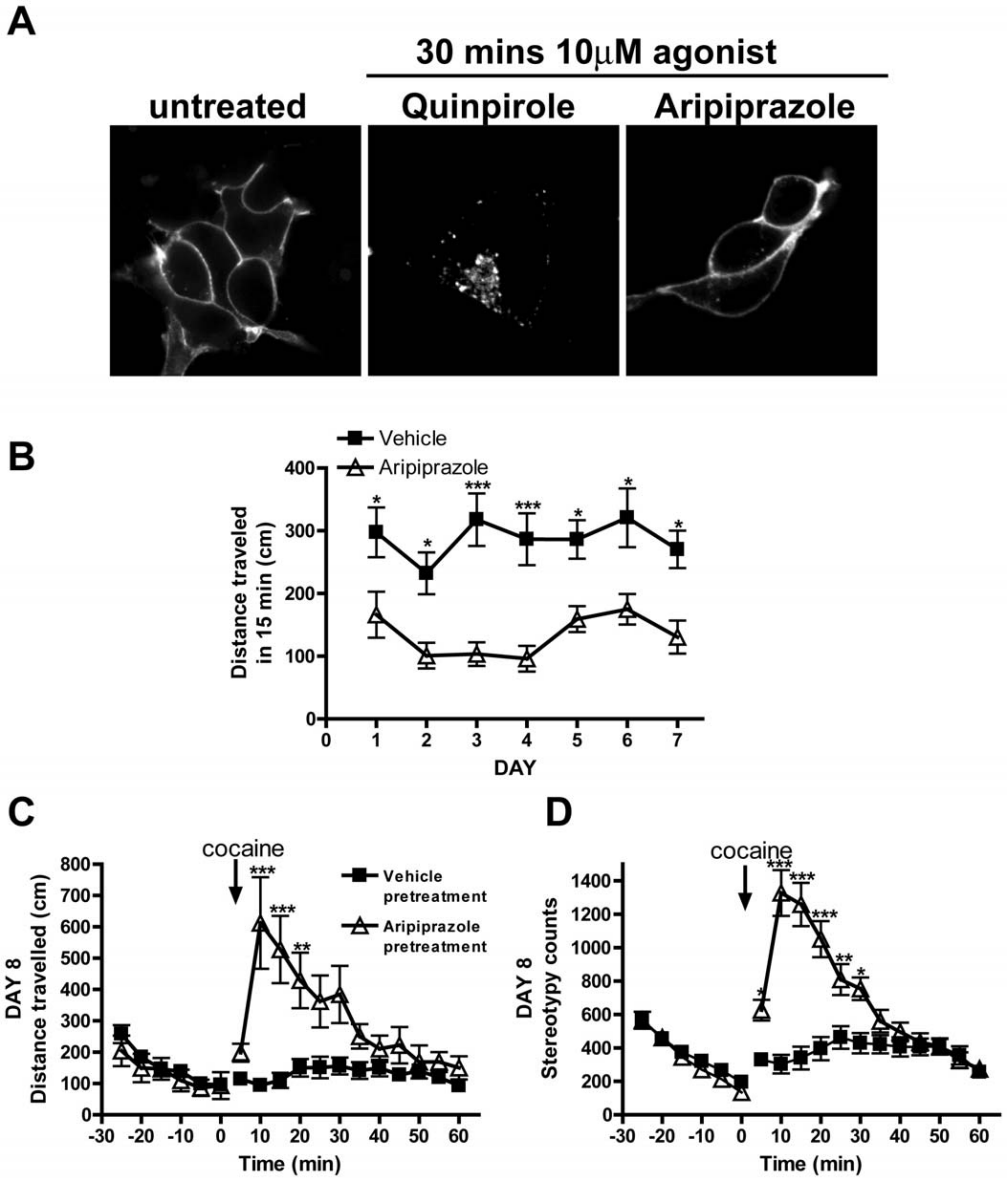
Repeated aripiprazole treatment augmented cocaine-induced behavioral responding. **A.** Aripiprazole does not induce endocytosis of D2Rs. HEK 293 cells stably expressing N-terminal FLAG-tagged D2Rs were incubated with M1 antibody to the extracellular tag for 20 mins then stimulated with quinpirole or aripiprazole (10 µM), or left untreated for 30 mins. Cells were fixed, blocked, permeabilised and receptors visualized with fluorescent secondary antibody Alexa 488. Quinpirole but not aripiprazole induced endocytosis of the D2R. **B.** Acute aripiprazole inhibits locomotion. WT C57/BL6 mice were injected daily (IP) with vehicle (n = 24) or aripiprazole (5 mg/kg, n = 24) and their locomotor activity recorded once per day for 7 days. Aripiprazole reduced locomotor activity when compared to vehicle treated animals across all 7 days (*p<0.05, ***p<0.001) and there was no significant change in the effect of aripiprazole and any day compared to day 1. **C, D.** Repeated aripiprazole pretreatment augments locomotor response to acute cocaine. On day 8, mice from (**B**) were injected with cocaine (IP, 20 mg/kg) and their locomotor activity (**D**) and stereotypy (**E**) recorded. Mice pretreated with repeated aripiprazole showed enhanced locomotor and stereotypic responses to acute cocaine compared to saline treated mice (***p<0.001, **p<0.01 and *p<0.05). Data was analyzed by either one or two-way ANOVA with Bonferroni multiple comparison tests and presented as mean ± S.E.M.

In agreement with previous studies, aripiprazole did not promote endocytosis of D2Rs *in vitro* [21], whereas quinpirole did [10] (Fig. 5A). WT mice were treated with vehicle or with aripiprazole (5 mg/kg) for 7 days. On day 1, aripiprazole decreased the locomotor responses of WT mice when compared to vehicle treated mice (Fig. 5B). In contrast to quinpirole (Fig. 4B), aripiprazole maintained locomotor inhibition across all days (Fig. 5B) (***p<0.001 and *p<0.01, saline treated mice versus aripiprazole treated mice), and showed no significant changes in locomotor inhibition across days compared to aripiprazole treatment on day 1. Hence, mice became tolerant to the locomotor inhibitory effects of quinpirole (Fig. 4B) but not aripriprizole (Fig. 5B).

On day 8, all mice were injected with cocaine (IP, 20 mg/kg). Mice pretreated with aripiprazole (Fig. 5C) showed similar increases in the locomotor responses to acute cocaine as mice pretreated with quinpirole (Fig. 4C). Aripiprizole pretreated mice showed significantly greater cocaine-induced locomotor activity (Fig. 5C) and stereotypic behavior (Fig. 5D) compared to mice pretreated with vehicle (***p<0.001 and **p<0.01). This was unexpected, since D2Rs neither recruit arrestins nor internalize in response to aripiprazole *in vitro* [20], [21], and we predicted that the failure of the mice to become tolerant to the locomotor inhibitory effects of aripiprizole (Fig. 5B) reflected that D2Rs had not been downregulated *in vivo*.

Indeed, radioligand binding in striatal membranes obtained from the mice in Figure 5 after day 8, demonstrated that aripiprazole pretreatment promoted no significant downregulation of D2Rs (Table 2) as expected. However, aripiprazole treatment did produce an unexpected upregulation of D1Rs changing the D2R∶D1R from 0.99 to 0.63, which was comparable to the change in ratio seen with quinpirole pretreatment (Table 2, *p<0.05, aripiprazole pretreated versus vehicle pretreated).

## Discussion

Receptor downregulation has long been implicated in adaptive responses to continued pharmacological stimuli. Here, we demonstrate that preventing GASP-1-mediated downregulation of D2Rs is associated with delays in the development of sensitization to the locomotor activating effects of cocaine. In addition, we show that downregulation of D2Rs, or upregulation of D1Rs via repeated dopamine receptor agonist treatment, significantly augments the acute locomotor response to acute cocaine exposure. Thus, we propose sensitization to cocaine reflects a change in the balance of D1-like versus D2-like dopaminergic signaling in favour of the stimulatory D1-G_s/olf_ pathway. Sensitization is thought to underlie at least some aspects of addiction [12], [14]. Thus, our hypothesis is consistent with reports that reduced signaling through G_i/o_ is a contributing factor in cocaine addiction [23]–[25]. However, while we favor this hypothesis, we cannot rule out that the three independent manipulations utilized here (disruption of GASP-1, treatment with quinpirole, treatment with aripiprizole), each of which affects the ratio of D2R∶D1R, is influencing sensitization to cocaine by a mechanism(s) other than altering this ratio.

Systemic D1R antagonists block both the development [26] and the expression [27], [28] of sensitization. Hence, D1Rs, due to their coupling to G_s/olf_, could possibly mediate some of the locomotor stimulating effects of dopamine. Indeed, acute, D1R-selective agonists promote locomotor activity [29] while D2R-selective agonists inhibit locomotor activity[30]. Therefore, under conditions of normal dopamine tone, the stimulatory effects of dopamine at the D1-G_s/olf_-coupled receptors are in balance with the inhibitory effects of D2-G_i_-coupled receptors. Under these conditions, the D1-like receptors (D1R and D5R) would be expected to endocytose and recycle, while the D2-like receptors (D2R, D3R and D4R) would be expected to show very little endocytosis, due to low receptor occupancy. However, under conditions of high dopamine tone (such as during cocaine-mediated blockade of dopamine reuptake), the occupancy, and therefore endocytic rate of all receptors would increase. Subsequently, endocytosed D1Rs would continue to recycle, but endocytosed D2Rs would be targeted for degradation via GASP-1. Consequently, the ratio of D1R versus D2R would be shifted towards D1R, especially under conditions of high D2R occupancy.

Antagonists at the D2R would be expected to prevent downregulation of D2Rs by preventing their internalization in response to dopamine [10], [31]. Consistent with this hypothesis, D2R antagonists block the development [16] but not the expression [13] of cocaine sensitization. This change in the balance of D1R and D2R signaling is only one of a plethora of changes that have been shown to occur during repeated drug use. For example, cocaine also increases extracellular glutamate levels. Interestingly, cocaine-induced increases in glutamate appear to be dependent on D1R action [32], [33], placing this change downstream of the activity at the dopamine receptors.

Recently it was reported that GASP-1 may be involved in the recycling rather than degradation of the D2R, since not only WT but also GASP-1 KO mice showed reduced D2R number following cocaine self-administration [34]. The reason for this difference remains unclear but could be due to several factors. First, our mice were generated in a pure C57Bl/6J background while the mice in [34] are on a mixed 129S2/SvPas/C57Bl/6J background. Hence, variation in genetic background among the mice in could account for differences in D1 and/or D2 receptor numbers. Furthermore, in [34], mice were sacrificed 60 minutes after the last injection of cocaine rather then 24 hours later as in our experiments. Therefore, the possibility remains that the reduction in D2R number reported in mice self-administering cocaine could be due to receptors having been endocytosed (and thus lost from the membrane preparation) but not yet recycled in the GASP-1 KO mice. Furthermore, spiperone, which was used as the radioligand in [34] recognizes D2R and D4R to a similar extent (Ki 0.06 nM and 0.08 nM respectively) but also has affinity at 5-HT receptors, while raclopride (used in this study) shows increased specificity for D2Rs over D3Rs and D4Rs (Ki 1.8 nM, 3.5 nM and 2400 nM respectively), and little affinity at 5-HT receptors. In addition, substantial downregulation of D2Rs was observed in [34] only in GASP-1 KO mice who had learned to self-administer cocaine, not during sensitization. Currently, it is unknown whether the learning necessary to acquire this task alters the levels of striatal D1 and D2Rs.

Importantly, *in vitro*, GASP-1 specifically binds to GPCRs that show pronounced proteolysis following prolonged agonist treatment (i.e. DOR [11], CB1 [35], bradykinin B1R ([36]) and D2R [10]). In addition, the inhibition of GASP-1 function by either over expression of the dominant negative version of GASP-1, cGASP, or interfering with the interaction of D2R with GASP using an inhibitory antibody, facilitated recycling and recovery of D2R responses rather than enhanced downregulation [10]. Together these data suggest that GASP-1 contributes to receptor degradation. Perhaps surprisingly, we found that GASP-1 KO mice show lower (rather than higher) baseline levels of D2Rs. These lower levels of D2R (and thus a lower baseline ratio of D2R∶D1R) did not significantly affect acute locomotor responses to cocaine, suggesting that it is the net change in the ratio of D2R∶D1R rather than the absolute ratio that is associated with a sensitized locomoter response. Intriguingly, we recently found that baseline levels of the CB1 cannabinoid receptor, which is also targeted for degradation by GASP-1 [35], were also lower in GASP-1 KO mice [37]. Thus, we propose that other cellular mechanisms that regulate receptor expression levels, perhaps decreased synthesis or transport of new receptors, may compensate for loss of GASP-1 in the GASP1 KO mice.

In conclusion, here we show that GASP-1-mediated degradation of D2Rs may be a contributing factor to cocaine-induced behavioral sensitization and that the ratio of D1R to D2R correlates with a change in behavioral locomotor response to acute cocaine. Downregulation of D2Rs has been implicated in the pathology of multiple neuropsychiatric disorders, and more than 50 drugs that target members of the dopamine receptor family have been developed as pharmacological interventions. Importantly, for the vast majority of these ligands, the effects of the drugs on the endocytic and postendocytic trafficking of the five distinct dopamine receptors remains undetermined. Our studies here clearly demonstrate that receptor trafficking can affect behavioral outcomes in rodent models of drug-induced behavioral plasticity, and suggest that an understanding of receptor trafficking could provide novel insight into the therapeutic utility of future pharmacological therapies.

## Materials and Methods

### Animals

Mice were housed according to sex and genotype, 4 per cage and maintained on a 12 hour light∶dark cycle (lights on at 7:00am) with continuous access to food and water.

### Drugs

Cocaine hydrochloride and (-)-quinpirole hydrochloride were purchased from Sigma Aldrich (St Louis, MO) and diluted in isotonic saline (0.9% sodium chloride). Aripiprazole was diluted in saline containing 1% Tween 20. All drugs were administered intraperitoneally (IP).

### Generation of GASP-1 Knockout mice

A targeting vector containing a neomycin resistance gene flanked by lox P Cre-recombination sites were inserted into the intron downstream of the GASP open reading frame (ORF) (intron 5). A third lox P site was inserted in the intron upstream of the GASP ORF. 30 µg of Not I-linearized knock-out (KO) vector DNA was electroporated into ∼10^7^ C57Bl/6 embryonic stem (ES) cells and selected with 200 µg/ml G418. Primary ES screening was performed by Southern blotting. The selected homologous recombinants were further transfected with a vector encoding Cre-recombinase to selectively excise the GASP-1 gene. ES cells deficient in GASP-1 were identified and blastocysts from these cells implanted into C57Bl/6J females.

### Locomotor Activity

Locomotor activity was monitored in an open field box (20 cm ×20 cm ×20 cm) with a Digiscan analyser (Accuscan Instruments Inc, Columbus, OH) where distance travelled was determined by consecutive breaking of adjacent photo beams. Stereotypy was estimated as repeated breaking of the same photocell beam. Animals were habituated to the open field for 30 min prior to injection and their activity recorded for 60 min post-drug treatment. Animals received saline injections (ml/kg) for 2 days prior to the start of drug or vehicle treatment to habituate them to handling and injection procedures.

### GASP detection in brain lysates

Brain lysates from wild type (WT), heterozygous (HET) and KO mice were separated on a 4–20% gradient Tris·HCl precast gel (Invitrogen, Carlsbad, CA) and transferred to 0.22 µm nitrocellulose at 200 mA for 4 h. Blots were cut below the 75-kDa marker band and were separately probed for β-Actin (lower blot) or GASP (upper blot). GASP blots were incubated for 1 h with rabbit anti-GASP (1∶1,000, 5% milk, [10]) and for 1 h with HRP-conjugated anti-rabbit secondary antibody (NEB, Ipswich, MA) (1∶4,000, 5% milk (Carnation, Nestle, Switzerland), 1 h at room temperature), then visualized with ECL plus (Amersham Pharmacia). β-Actin blots were incubated for 1 h with mouse anti- β-Actin (1∶10,000, 5% milk) and for 1 h with HRP-conjugated anti-mouse secondary antibody (1∶4,000, 5% milk at room temperature) and visualized with ECL plus.

### Radioligand binding

24 hours after the last drug administration, mice were sacrificed by cervical dislocation and their striatum harvested for analysis of D1R and D2R. Striatal tissue from 4–8 mice were pooled and homogenized in 0.32M ice cold sucrose. Samples were centrifuged at 40,000rpm for 20 min and resuspended in 0.32M ice cold sucrose. This step was repeated twice, and the final pellet frozen at −20°C until use. Protein concentration was determined by Bio-Rad protein assay (Bio-Rad, Hercules, CA).

D1R number was measured using [^3^H]-*N*-methyl-SCH23390 ([^3^H]-SCH23390, 85 Ci/mmol, Perkin Elmer, Boston, MA). Total homogenates corresponding to 20 µg protein were prepared in binding buffer containing 50 mM Tris-HCl pH 7.4, 1 mM CaCl_2_, 5 mM MgCl_2_, 5 mM KCl, 120 mM NaCl and 0.078 nM–20 nM [^3^H]-SCH23390 in a final volume of 200 µl. Samples were incubated for 60 minutes at 37°C and filtered through Whatman GF/C filters that had been pretreated with 0.3% Poly(ethyleneimine) (PEI, Sigma). The filters were washed three times in ice cold binding buffer and dried overnight at room temperature. The filters were then incubated overnight in 50 µl of scintillation fluid (Microscint 20, Perkin Elmer) prior to counting in a Packard cell top scintillation counter (Perkin Elmer). Specific binding was calculated as total minus nonspecific binding performed in the presence of cold SCH23390 (20 µM). D2R number was determined using [^3^H]-Methoxy-raclopride ([^3^H] -Raclopride 62.2 Ci/mmol, from Perkin Elmer) and binding performed as for the D1R. Nonspecific binding was determined in the presence of 20 µM cold raclopride.

### Cell culture and Immunocytochemistry

Human embryonic kidney (HEK) 293 cells (American Type Culture Collection) were grown in DMEM (Invitrogen) supplemented with 10% fetal bovine serum (HyClone). N-terminal FLAG-tagged D2Rs were stably expressed in HEK293 cells. For generation of clonal stable cell lines, single colonies were chosen and propagated in the presence of selection-containing media. For immunocytochemistry experiments, cells stably expressing FLAG-tagged D2Rs were grown on coverslips to 50% confluency. Live cells were fed M1 antibody (Sigma) directed against the FLAG tag (1∶1,000, 30 min). Cells were then treated with agonist (10 µM quinpirole or 10 µM aripiprazole) for 30 min or left untreated. Cells were then fixed with 4% formaldehyde in PBS. After fixation, cells were permeabilized in blotto with 0.1% Triton X-100 and stained with fluorescently conjugated secondary antibody (1∶500, Molecular Probes).

### Statistical Analyses

Data were analyzed using one or two way analysis of variance (ANOVA) followed by Bonferroni multiple comparisons ± S.E.M.
